# Preparation and Characterization of Thermo-Responsive Rod-Coil Diblock Copolymers

**DOI:** 10.3390/polym9080340

**Published:** 2017-08-04

**Authors:** Yang-Yen Yu, Wen-Chen Chien, Chia-Liang Tsai

**Affiliations:** 1 Department of Materials Engineering, Ming Chi University of Technology, No. 84, Gongzhuan Rd., Taishan Dist., New Taipei City 24301, Taiwan; amyyu1005@gmail.com; 2 Department of Chemical and Materials Engineering, Chang Gung University, No. 259, Wenhua 1st Rd., Guishan Dist., Taoyuan City 33302, Taiwan; 3 Department of Chemical Engineering, Ming Chi University of Technology, No. 84, Gongzhuan Rd., Taishan Dist., New Taipei City 24301, Taiwan; wcchien@mail.mcut.edu.tw

**Keywords:** atom transfer radical polymerization, Rod-coil diblock copolymers, Thermo-reversible polymers

## Abstract

In this study, we synthesized amphiphilic poly(2,7–(9,9–dioctylfluorene))–*block–N*,*N*–(diisopropylamino)ethyl methacrylate (POF–*b*–PDPMAEMA) rod-coil diblock copolymers by atom transfer radical polymerization (ATRP). The structure and multifunctional sensing properties of these copolymers were also investigated. The POF rod segment length of 10 was fixed and the PDPAEMA coil segment lengths of 90 and 197 were changed, respectively. The micellar aggregates of POF_10_–*b*–PDPAEMA_90_ rod-coil diblock copolymer in water showed a reversible shape transition from cylinder bundles to spheres when the temperature was changed from 20 to 80 °C or the pH was changed from 11 to 2. The atomic force microscopy (AFM) and transmission electron microscopy (TEM) measurements indicated that the temperature had also an obvious influence on the micelle size. In addition, since POF_10_–*b*–PDPAEMA_90_ had a lower critical solution temperature, its photoluminescence (PL) intensity in water is thermoreversible. The PL spectra showed that the POF–*b*–PDPAEMA copolymer had a reversible on/off profile at elevated temperatures, and thus could be used as an on/off fluorescent indicator for temperature or pH. The fluorescence intensity distribution of pH switched from “off–on” to “on–off” as the temperature increased. These results showed that the POF–*b–*PDPAEMA copolymer has a potential application for temperature and pH sensing materials.

## 1. Introduction

The self-assembly of conjugated rod-coil block copolymers has been studied extensively towards novel structural, functional, and physical properties of supramolecular species [1,2,3]. The aggregation and microphase separation of such block copolymers driven by various novel functionalities of p–p interaction through solvent selection or rigid segmentation may result in various nanosized morphologies [4,5,6]. Different conjugated rod-coil block copolymers have been reported in the literature, including those of thiophene [7,8], phenylene [9,10,11], and quinolone [12,13]. The fluorene-based rod-coil block copolymers were used in this study because of their high thermal/chemical stability and good fluorescence quantum yield [14,15,16]. Incorporating the coil segment into the backbone not only allows the possibility to control the electronic and optoelectronic properties, but also produces self-assembled nanostructures, such as spheres, worms, honeycombs, or linear structures [17]. Besides, the combination of the stimulus-responsive coil section and the tunable photophysical properties will result in novel multifunctional sensing materials [18,19]. Recently, the studies of synthesis, self-assembly of a dual thermal and pH-responsive copolymer have been reported in the literature [20,21]. The micellar morphology of PDMAEMA-based coil-coil block copolymers has been also applied to biology [22,23,24,25]. The aggregation of rod-coil block copolymers in water and THF mixed solvents caused a reduction of quantum efficiency due to the formation of excimers [26,27,28,29,30]. However, the effects of detailed structure of rod-coil block copolymers on the multifunctional sensing characteristics and photophysical properties also needs to be explored further.

Two kinds of amphiphilic POF–*b*–PDPAEMA rod-coil diblock copolymers with different segment lengths, POF_10_–*b–*PDPAEMA_90_ and POF_10_–*b*–PDPAEMA_197_, were synthesized by atom transfer radical polymerization (ATRP) for systematic investigation in this study. Some important parameters such as solution temperature, acid-base value, and water/tetrahydrofuran ratio were used to study the surface morphology and photophysical properties of copolymers. Micellar aggregation was investigated by atomic force microscopy (AFM) and transmission electron microscopy (TEM) measurements. The photophysical properties of amphiphilic rod-coil diblock copolymer in solution were discussed by UV–Vis absorption spectra and photoluminescence (PL). The surface structure and photophysical properties of rod-coil block copolymers are related to rod/coil ratios and stimuli parameters. The results show that the surface structure and fluorescence characteristics for the amphiphilic POF–*b*–PDPAEMA rod-coil diblock copolymers can be manipulated by tuning the mixed solvent composition, temperature, and pH.

## 2. Experimental

*N,N*–(Diisopropylamino) ethyl methacrylate (DPAEMA, Aldrich, 98%, St. Louis, MO, USA) was purified on an aluminium oxide column. 1,1,4,7,7–Pentamethyldiethyl-enetriamine (Aldrich, 99%), Copper(I) bromide (Aldrich, 99.99%), 9,9–Dioctyl–2,7–dibromofluorene (Aldrich, 96%), 2–bromoisobutyryl bromide, 4–bromobenzyl alcohol, anisole, sodium carbonate, tetrakis (triphenylphosphine)palladium, and *N,N*–dimethylacetamide sodium carbonate were used without purification. (9,9–dioctylfluorene)–block–poly(2,7–(9,9–dioctylfluorene))–*block*–*N*,*N*–(Diisopropylamino)ethyl methacrylate) (POF–*b–*PDPAEMA) amphiphilic copolymer was prepared by atom transfer radical polymerization.

First, POF–Br was prepared by Suzuki coupling [31,32]. The prepared POF–Br solid was redissolved in a THF solvent and then precipitated for purification by using methanol to obtain purer POF–Br (*M*_n_ = 3240 and PDI = 1.31). The preparation of POF_10_–*b*–PDPAEMA_90_ is described below. Macroinitiator, PMDETA, CuBr, and *N,N*–(diisopropylamino)ethyl methacrylate (DPAEMA at a mole ratio of 1:1:1:267 were mixed with an appropriate amount of anisole solvent. The mixture was stirred homogeneously at room temperature for 1 h, and then temperature was raised to 90 °C for 5.5 h. Then, the obtained solution was dried in vacuum at 50 °C to obtain PF_10_–*b*–PDPAEMA_90_（*M*_n_ = 19,200 and PDI = 1.29）as a brown solid (S1). The result of ^1^H NMR (CDCl_3,_ δ(ppm)) for the POF–*b*–PDPAEMA copolymer was as follows: 0.81–0.93 (3H, –CH_2_C(CH_3_)–), 1.69–1.89 (2H, –CH_2_C(CH_3_)–), 2.32–2.43 (14H, –N(C_3_H_7_)_2_), 2.55 (2H, –OCH_2_CH_2_N(C_3_H_7_)_2_), 4.05 (2H, –OCH_2_CH_2_N–(C_3_H_7_)_2_), 7.45–7.90 (10H, POF and phenyl aromatic H). POF–*b*–PDPAEMA micelles were prepared in different ratios of solutions. The preparation of POF–*b*–PDPAEMA micelles using different solvent compositions is described below. POF–*b*–PDPAEMA amphiphilic copolymer was dissolved in THF. Then, MeOH was slowly added into solution to obtain a THF/MeOH mixed solvent solution with a desired MeOH/THF ratio, 10, 25, 50, 75, and 90 wt %, respectively. The concentration of POF–*b–*PDPAEMA copolymer in mixed solution was maintained at 1 mg/mL. To study the thermo-responsive behavior, the POF–*b*–PDPAEMA amphiphilic copolymer solution in MeOH (1 mg/mL) was heated to 30, 40, 50, 60, or 80 °C, respectively, and maintained temperature for 50 h to reach the thermodynamic equilibrium. On the other hand, the pH of POF–*b–*PDEAEMA amphiphilic copolymer solution was adjusting between 2 and 9 by using HCl and NaOH for different pH behavior studies. In each run, the solution had to stand for 50 h to reach equilibrium before measurement.

The chemical structure of synthesized copolymers was confirmed by Fourier transform infrared (FTIR) spectra (PerkinElmer Spectrum spectrophotometer, Waltham, MA, USA) and NMR spectra (Jeol EX-400 spectrometer, Tokyo, Japan). The photophysical properties of copolymer films were tested by ultraviolet-visible spectrophotometry (Jasco Model V-650, Oklahoma, OK, USA) and photoluminescence spectroscopy (Horiba Jobin Yvon Fluoromax-4 Spectro fluorometer, Tokyo, Japan). The adsorption spectra were measured in the range of 200–800 nm and an excitation wavelength of 450 nm was used in emission spectrum. Gel permeation chromatography calibrated by 0.5 wt % PS standard in THF with a flowrate of 1 mL/min at 40 °C was used to measure the molecular weight distribution of copolymers. A refractive index detector (Schambeck SFD GmbH, model RI 2000, Bad Honnef, Germany) with a PLgel 5 μm mixed-C and D column were used in gel permeation chromatography. Surface structure of copolymers were examined by a transmission electron microscopy (JEOL 1210) and atomic force microscope (Bruker Nanoscope DI III multimode AFM, Billerica, MA, USA) measurements under an acceleration voltage of 100 kV and a tapping mode, respectively. 

## 3. Results and Discussion

**Figure 1:**
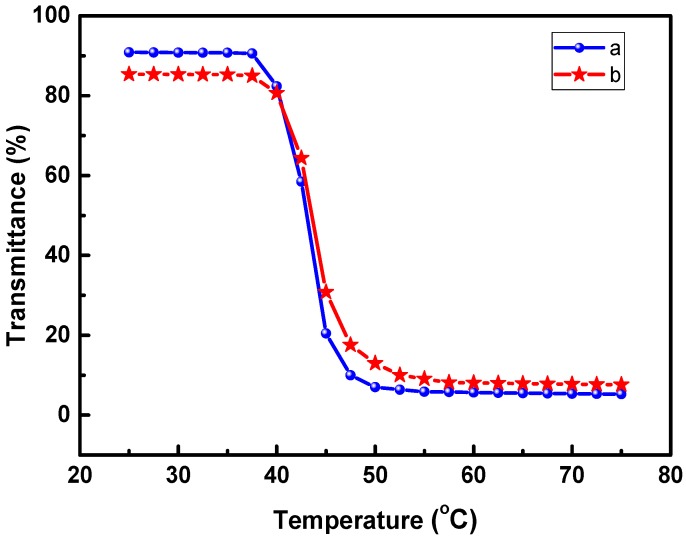
Variation of optical transmittance with temperature (30–80 °C): (**a**) POF_10_–*b–*PDPAEMA_197_ and (**b**) POF_10_–*b–*PDPAEMA_90_.

Figure 1 illustrates the variation of optical transmittance with temperature for the prepared copolymers, POF_10_–*b–*PDPAEMA_197_ and POF_10_–*b*–PDPAEMA_90_. It shows that the copolymer with a longer PDEAEMA chain length had a high transmission because of favorable solubility in water. In addition, the copolymer exhibited a lower critical solution temperatures (LCST) of 40 °C. At this temperature, the optical transmission of the copolymer solution decreased, because the micelle structure of copolymers aggregated, leading to reduced transmittance. The optical transmittance decreased as the chain length of the DPAEMA segment increased. Therefore, the reduction of optical transmittance for POF_10_–*b–*PDPAEMA_197_ was more apparent than that obtained from POF_10_–*b–*PDPAEMA_90_.

**Figure 2:**
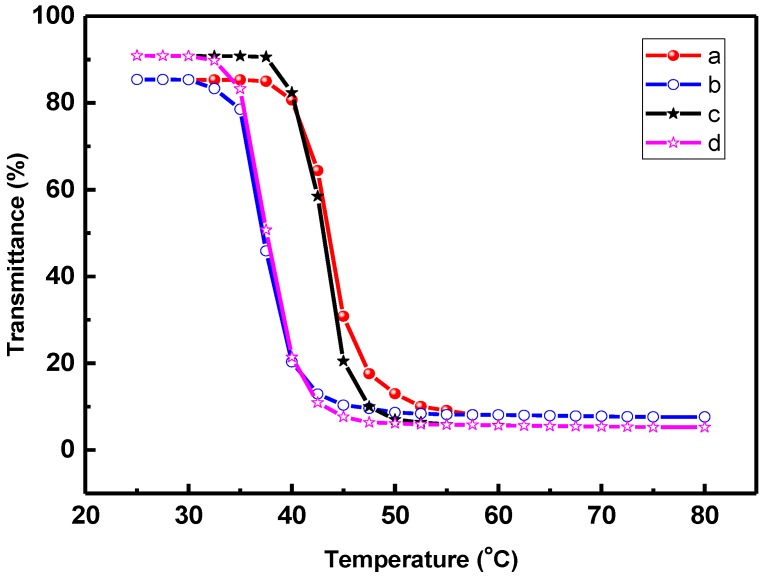
Variation of optical transmittance of (**a**,**b**) POF_10_–*b–*PDPAEMA_90_ and (**c**,**d**) POF_10_–*b–*PDPAEMA_197_ in water at a heating (**a**,**c**)–cooling (**b**,**d**) cycle between 30–80 °C.

Figure 2 illustrates the variation of optical transmittance of POF_10_–*b*–PDPAEMA_90_ and POF_10_–*b*–PDPAEMA_197_ in water when subjected to a heating (curve a, c)–cooling (curve b, d) cycle between 30 and 80 °C, respectively. It shows the individual heating and cooling curves form a loop for POF_10_–*b–*PDPAEMA_90_ and POF_10_–*b–*PDPAEMA_197_, respectively. This hysteresis effect results from the fact that intra-chain and inter-chain hydrogen bonding forms when different segments in the copolymers aggregate together.

Such a thermo-responsive behaviors of copolymers have been reported [33]. The temperature-sensitive segment of PF_10_–*b–*PDPAEMA_197_ is longer, so its shape changes rapidly as the temperature changes, resulting in a larger slope of the change in optical transmittance and thus a narrower loop width than the PF_10_–*b–*PDPAEMA_90_ [34].

**Figure 3:**
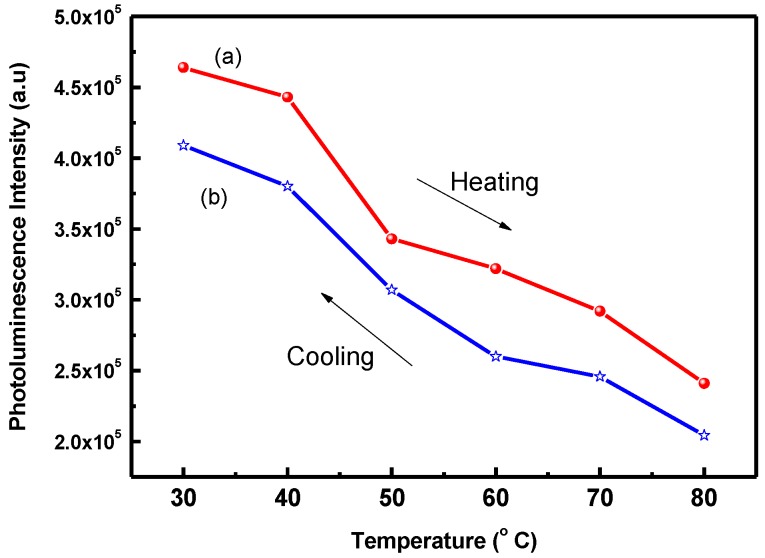
Photoluminescence intensity of POF_10_–*b–*PDPAEMA_90_ in water at a heating–cooling cycle between 30 and 80 °C: (**a**) heating (**b**) cooling.

Figure 3 illustrates the PL intensity of POF_10_–*b*–PDPAEMA_90_ in water when it was subjected to a heating and cooling cycle between 30 and 80 °C. It shows that the PL intensity decreased at the heating stage and increased again at the cooling stage. The formation of intramolecular hydrogen bonding between PDPAEMA chains results in this thermo-responsive phenomenon. Heating the solution above the LCST (40 °C) caused the intramolecular hydrogen bonding of the PDPAEMA and the structure formed closely packed aggregates that resulted in the reduction of PL intensity [24,33]. On the contrary, cooling the solution below the LCST led to a swelling of the PDPAEMA corona in water. Consequently, the POF core can absorb more incident light and increases the light emission intensity.

**Figure 4:**
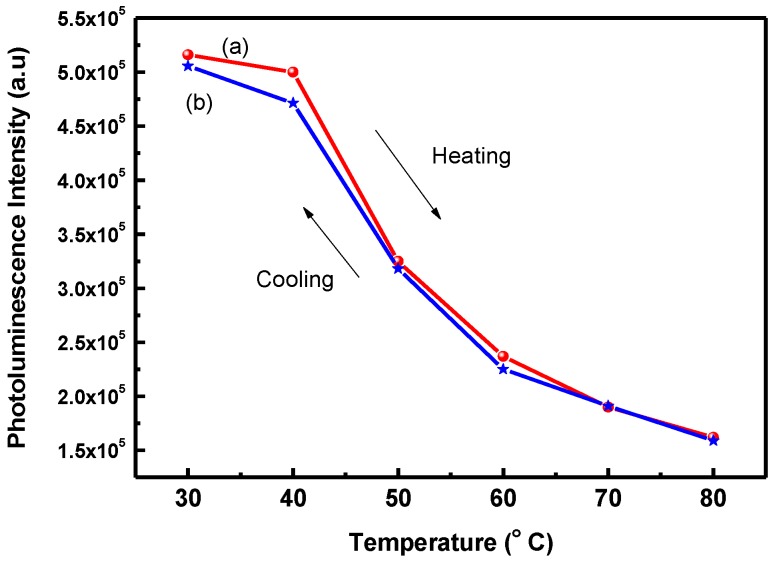
Photoluminescence intensity of POF_10_–*b*–PDPAEMA_197_ in water at a heating–cooling cycle between 30 and 80 °C: (**a**) heating (**b**) cooling.

Figure 4 illustrates the PL intensity of POF_10_–*b–*PDPAEMA_197_ in water when it was subjected to a heating and cooling cycle between 30 and 80 °C. Comparing Figure 3 and Figure 4 shows that the change of fluorescence intensity for POF_10_–*b–*PDPAEMA_197_ is significantly faster than POF_10_–*b–*PDPAEMA_90_, because POF_10_–*b–*PDPAEMA_197_ has longer thermo-responsive chain lengths that caused a faster aggregation of micelles and thus the apparent change in photoluminescence.

**Figure 5:**
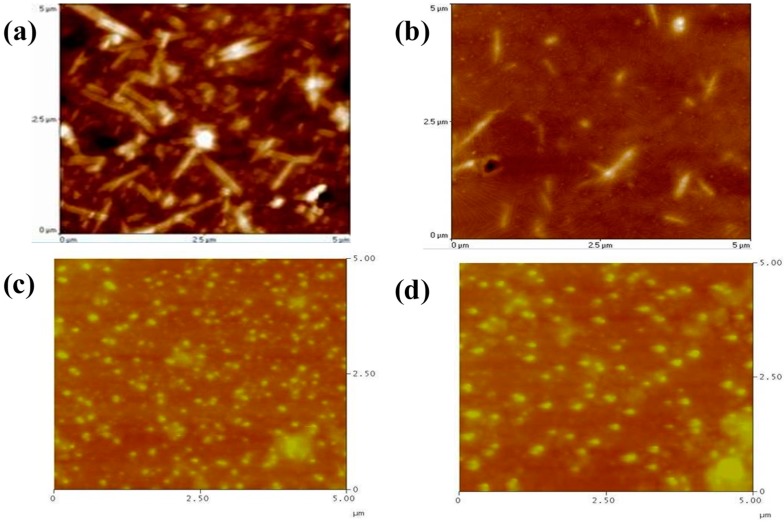
AFM images of POF_10_–*b–*PDPAEMA_90_ aggregates in water at temperatures of (**a**) 30 °C, (**b**) 50 °C, (**c**) 60 °C, (**d**) 80 °C.

Figure 5 illustrates the variation in the surface structures for the POF_10_–*b–*PDPAEMA_90_ in water with temperature. It shows that the surface structure consisted of cylinder bundles at 30 °C (below the LCST), but changed to cylinders at 50 °C (above the LCST). However, the surface structure exhibited spheres at 60 °C, and the spheres aggregated at 80 °C. The described structural change of the micelles can be attributed to the variation in the hydrophilic/hydrophobic property of PDEAEMA. The highly hydrophilic PDPAEMA block showed an extended corona conformation as the temperature was below its LCST. However, as the temperature was raised above the LCST, the formation of intramolecular hydrogen bonding between the PDPAEMA chains caused a closely packed arrangement, thus water molecules were excluded from the corona.

**Figure 6:**
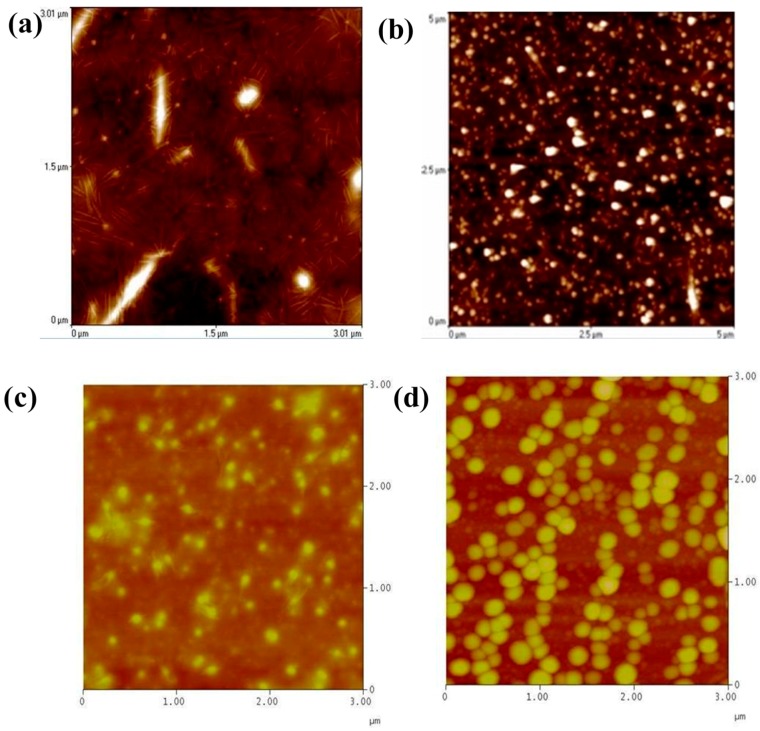
AFM images of POF_10_–*b–*PDPAEMA_197_ aggregates in water at temperatures of (**a**) 30 °C, (**b**) 50 °C, (**c**) 60 °C, (**d**) 80 °C.

Therefore, the radius of cylindrical micelles became smaller. Nevertheless, increasing the temperature from 60 to 80 °C could enhance the intramolecular hydrogen bonding of the corona; thus, the corona became highly contracted, disrupting the POF aggregation. Figure 6 shows the variation in the surface structures for the POF_10_–*b–*PDPAEMA_197_ in water with temperature. The surface structure consisted of cylinder bundles at 30 °C, but changed to spheres at 50 °C (above the LCST) because of the longer chain length of the hydrophobic PDPAEMA segment [35]. Aggregates of spherical micelles were observed at higher temperatures (60–80 °C) because the long hydrophobic segment reduced the colloidal stability in water, resulting in larger interconnected micelles, as shown in Figure 6c,d.

**Figure 7:**
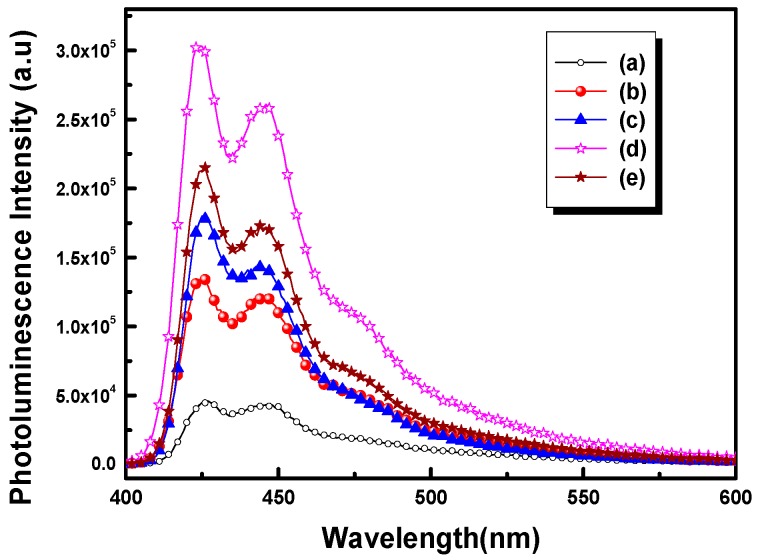
Photoluminescence spectra of POF_10_–*b–*PDPAEMA_90_ in water at different pH levels: (**a**) 2, (**b**) 5, (**c**) 7, (**d**) 9, (**e**) 11.

**Figure 8:**
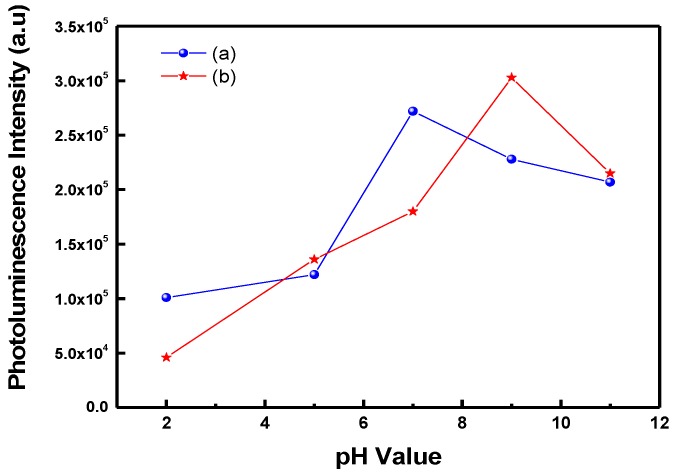
Variation of photoluminescence intensity of (**a**) POF_10_–*b*–PDPAEMA_197_ and (**b**) POF_10_–*b*–PDPAEMA_90_ with different pH levels.

Figure 7 illustrates the PL spectra of POF_10_–*b–*PDPAEMA_90_ in water at different solution pH. It can be seen that the PL intensity was weak at acidic pH, but strengthened as the pH increased from pH = 2 to 9. Over pH = 9, the PL intensity decreased again with increasing pH. This is because the PDPAEMA chains at acidic pH led to the formation of large polymer spheres so that scarcely any incident light was absorbed by the POF core, resulting in weak emission intensity. In addition, the pH-sensitive PDPAEMA segment undergoes a decrease in the conjugate plane resulting from the aggregation of the deprotonated chains in the high pH environment, resulting in a decrease in the fluorescence intensity [36,37]. Figure 8 illustrates the PL intensity of POF_10_–*b–*PDPAEMA_90_ and POF_10_–*b–*PDPAEMA_197_ in water at different solution pH. It shows that the PL intensity of POF_10_–*b*–PDPAEMA_197_ decreased at lower pH levels (pH = 7) than that (pH = 9) of POF_10_–*b–*PDPAEMA_90_, because the copolymer with a longer PDPMAEMA segment more readily undergoes aggregation at lower pH levels.

**Figure 9:**
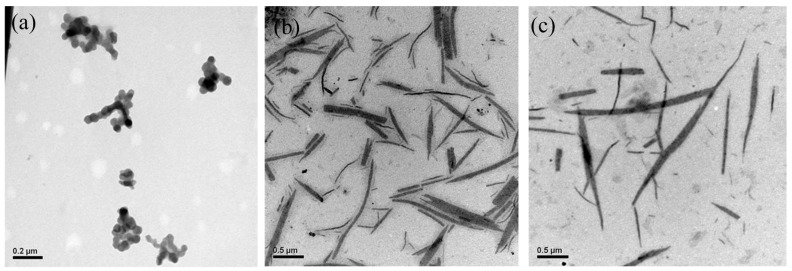
TEM images of POF_10_–*b–*PDPAEMA_90_ aggregates in water at different pH levels: (**a**) 2, (**b**) 7, (**c**) 11.

**Figure 10:**
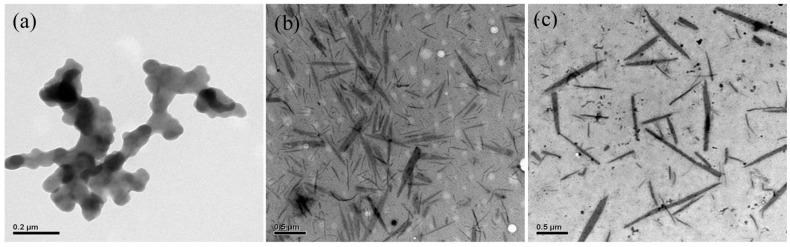
TEM images of POF_10_–*b–*PDPAEMA_197_ aggregates in water at different pH levels: (**a**) 2, (**b**) 7, (**c**) 11.

Figure 9 and Figure 10 illustrate the TEM images of the POF_10_–*b–*PDPAEMA_90_ and POF_10_–*b–*PDPAEMA_197_ micellar aggregates to show the structural change induced by pH variation. It shows that the spherical small micelles were observed at pH = 2, as shown in Figure 9a. As the pH increased to 7, the cylinder bundles were observed as shown in Figure 9b due to the interaction between the PDPAEMA and the POF π–π bond at both ends of the POF, the PDPAEMA is deprotonated and induces the aggregation, resulting in the transformation of the structure into shorter and coarse cylindrical micelles. As the pH value continues to rise, its morphology is transformed into a single monoclinic micelle, because the increase in the pH value suppresses the electrostatic repulsion of PDPAEMA, and the diameter becomes smaller into a thin cylindrical micelle as shown in Figure 9c. The result indicates that the pH value controls the degree of charge in the corona of the PDPAEMA block and induces swelling or shrinking because of the electrostatic repulsion. It means that the addition or removal of a proton (H) to or from PDPAEMA has an obvious influence on the copolymer structure. As shown in Figure 10, the POF_10_–*b–*PDPAEMA_197_ copolymers show a similar structural transformation.

## 4. Conclusions

In this study, we have synthesized the POF–*b–*PDPAEMA rod-coil block copolymers that show a significant variation in their surface structure and photophysical properties with respect to the temperature and pH. The micellar aggregates of POF_10_–*b–*PDPAEMA_90_ in water show a reversible change of surface structure from cylinder bundles to spheres under a heating–cooling cycle of 30–80 °C. The LCST of POF_10_–*b–*PDMAEMA_90_ decreased with an increase in solution pH values due to the deprotonation of the PDPAEMA block. On the other hand, the light emission intensity for POF_10_–*b*–PDPAEMA_90_ in water was thermo-responsive, depending on its lower critical solution temperatures. It means that the prepared copolymers in this study can be applied as on/off fluorescence indicators and thus as temperature and acid-base value sensory materials, since the POF–*b–*PDPAEMA copolymer shows a stable and reversible ‘‘on–off’’ profile.

## Supplementary Materials

Supplementary Materials are available online at www.mdpi.com/2073-4360/9/8/340/s1. The GPC curves could be found in Figure S1.
