# Supplementary material for: Preparation and Characterization of Thermo-Responsive Rod-Coil Diblock Copolymers
Source: Polymers (Basel). 2017 Aug 4;9(8):340. doi: 10.3390/polym9080340 (PMC6418999; doi:10.3390/polym9080340)
Supplement: Supplementary file 1 [file polymers-09-00340-s001.pdf]

polymers

MDPI

The supporting information

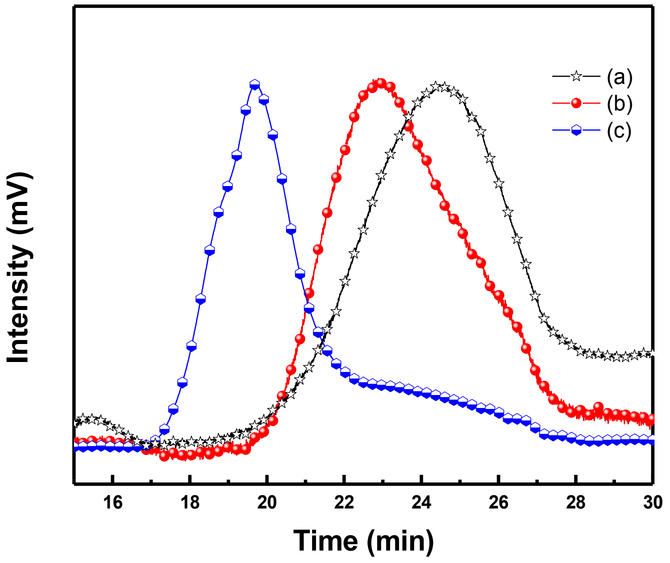


**Figure S1.** GPC curves of the prepared (a) $POF_{10}$; (b) $POF_{10}$-b-$PDMAEMA_{90}$ and (c) $POF_{10}$-b-$PDMAEMA_{197}$.
